# imPlatelet classifier: image‐converted RNA biomarker profiles enable blood‐based cancer diagnostics

**DOI:** 10.1002/1878-0261.13014

**Published:** 2021-06-20

**Authors:** Krzysztof Pastuszak, Anna Supernat, Myron G. Best, Sjors G.J.G. In 't Veld, Sylwia Łapińska‐Szumczyk, Anna Łojkowska, Robert Różański, Anna J. Żaczek, Jacek Jassem, Thomas Würdinger, Tomasz Stokowy

**Affiliations:** ^1^ Laboratory of Translational Oncology Intercollegiate Faculty of Biotechnology University of Gdańsk and Medical University of Gdańsk Poland; ^2^ Department of Algorithms and Systems Modelling Faculty of Electronics, Telecommunications and Informatics Gdańsk University of Technology Poland; ^3^ Department of Neurosurgery Amsterdam University Medical Center Vrije Universiteit Medical Center Cancer Center Amsterdam The Netherlands; ^4^ Brain Tumor Center Amsterdam Amsterdam University Medical Center Vrije Universiteit Medical Center Cancer Center Amsterdam The Netherlands; ^5^ Department of Pathology Amsterdam University Medical Center Vrije Universiteit Medical Center Cancer Center Amsterdam The Netherlands; ^6^ Department of Gynecology, Gynecological Oncology and Gynecological Endocrinology Medical University of Gdańsk Poland; ^7^ Department of Gynecology, Gynecological Oncology and Gynecological Endocrinology Medical University of Gdańsk Poland; ^8^ Department of Oncology and Radiotherapy Medical University of Gdańsk Poland; ^9^ Department of Clinical Science University of Bergen Norway

**Keywords:** image‐based classification, liquid biopsy, RNA sequencing, tumor‐educated platelets

## Abstract

Liquid biopsies offer a minimally invasive sample collection, outperforming traditional biopsies employed for cancer evaluation. The widely used material is blood, which is the source of tumor‐educated platelets. Here, we developed the imPlatelet classifier, which converts RNA‐sequenced platelet data into images in which each pixel corresponds to the expression level of a certain gene. Biological knowledge from the Kyoto Encyclopedia of Genes and Genomes was also implemented to improve accuracy. Images obtained from samples can then be compared against standard images for specific cancers to determine a diagnosis. We tested imPlatelet on a cohort of 401 non‐small cell lung cancer patients, 62 sarcoma patients, and 28 ovarian cancer patients. imPlatelet provided excellent discrimination between lung cancer cases and healthy controls, with accuracy equal to 1 in the independent dataset. When discriminating between noncancer cases and sarcoma or ovarian cancer patients, accuracy equaled 0.91 or 0.95, respectively, in the independent datasets. According to our knowledge, this is the first study implementing an image‐based deep‐learning approach combined with biological knowledge to classify human samples. The performance of imPlatelet considerably exceeds previously published methods and our own alternative attempts of sample discrimination. We show that the deep‐learning image‐based classifier accurately identifies cancer, even when a limited number of samples are available.

## Graphical abstract

To our knowledge, this is the first report that uses a deep neural network to analyze RNA‐sequencing data in liquid biopsies. imPlatelet method shows superior performance, detecting cases even in the early stage ovarian cancer. It shows remarkable potential in healthy individuals' indication. We believe that similar approach could be applied to sequencing data of tissues or single cells.

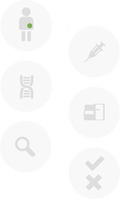

**Abbreviations**
AUC: area under curve
CTC: circulating tumor cells
ctDNA: circulating tumor DNA
DNN: deep neural network
KEGG: Kyoto Encyclopedia of Genes and Genomes
MUG: Medical University of Gdańsk
NSCLC: non‐small cell lung cancer
OC: ovarian cancer
PSO: particle swarm optimization
ROC: receiver operating characteristic
SVM: support vector machine
TEPs: tumor-educated platelets

## 1 Introduction

Sequencing technologies have enabled in‐depth analysis of liquid biopsies in cancer, offering a minimally invasive sample collection. This new approach to patient management has lately revolutionized cancer diagnostics, allowing for accurate detection of cancer [1], monitoring of therapy efficiency [1], tracking treatment resistance [2], or detecting minimal residual disease [3]. The most widely used material for liquid biopsy analysis is blood—the source of circulating tumor cells (CTCs) [4], circulating tumor DNA (ctDNA) [5], circulating microRNAs [6], extracellular vesicles [7], and, lately, tumor‐educated platelets (TEPs) [8].

Multiple nucleic acid‐based assays are applied to study liquid biopsy material [9, 10, 11, 12]. Since the introduction of high‐throughput analysis methods, data complexity and multitude of features requires more advanced approaches than assuming a simple cutoff for the interpretation of the final result. Machine learning has been used to classify RNA expression microarrays [13]. Later, more complex classifiers provided useful prognosis estimates [13, 14], classified malignant and benign tumors [15, 16], and helped to identify the primary site of the tumor [17]. Methods such as support vector machines (SVM), particle swarm optimization (PSO), random forests, or elastic nets in convolutional neural networks have been widely used in cancer research [18, 19, 20, 21]. The utility of SVM and PSO‐enhanced SVM, applied to spliced (intron‐spanning) platelet RNA reads, has been demonstrated for non‐small cell lung cancer (NSCLC), glioblastoma, colorectal cancer, pancreatic cancer, breast cancer, hepatobiliary cancer, multiple myeloma, and sarcoma [9, 15, 16, 17, 18]. However, further optimization of machine‐learning methodologies is required to improve detection accuracies.

As artificial intelligence includes multiple types of machine‐learning and deep‐learning approaches, we hypothesized that medical diagnostics could also benefit from this ongoing machine‐learning revolution. We observed that thromboSeq method, which relies on PSO‐enhanced SVM [22], might benefit from improvements in the aspect of balanced classification accuracy, the computational power needed, user experience, or execution time. Therefore, we developed an enhanced bioinformatics approach and termed the method imPlatelet. Among different types of data analysis and processing methods, we selected a training approach that works similarly to a human brain. When processing platelet transcriptomics data, we applied an image‐based classification which has lately introduced new levels of precision to fields other than medicine. In addition to creating pictures of platelet RNA reads, we also implemented biological knowledge from Kyoto Encyclopedia of Genes and Genomes (KEGG) database [23]. This enabled us to reach higher accuracies, even in a small cohort of patients.

First, we tested imPlatelet method on a published cohort of NSCLC patients, sarcoma patients, former sarcoma patients, and healthy donors [24, 25]. Next, we applied the developed tool to platelets collected from an independent cohort of patients, unpublished before. We focused on blood platelet RNA collected from patients with ovarian cancer (OC). We used blood samples collected from gynecological patients suffering from noncancer conditions, referred to as benign controls, and healthy donors as a frame of reference. Finally, to further validate the algorithm, we applied it to bulk RNA‐sequencing tissues deposited at The Cancer Genome Atlas. The newly developed classification was then compared with currently used thromboSeq method [22]. In this work, we show a deep‐learning image‐based classifier which identifies cancer cases very accurately.

## 2 Materials and methods

### 2.1 Blood samples

The detailed list of all cases, along with their characteristics, is presented in Table 1. Healthy control, NSCLC, and sarcoma samples were collected at VU University Medical Center (Amsterdam, the Netherlands), Netherlands Cancer Institute (Amsterdam, the Netherlands), and Massachusetts General Hospital (USA). Whole blood samples of OC patients were collected to 6 mL EDTA Vacutainer tubes at the Department of Gynecology, Gynecological Oncology and Gynecological Endocrinology, Medical University of Gdańsk (MUG). Benign control samples and OC samples were collected at MUG. The study was approved by the Independent Ethics Committee of the MUG (NKBBN/434/2017). All patients signed informed consent. Procedures involving human subjects were in accordance with the Helsinki Declaration, as revised in 1983.

**Table 1:** Demographic data of cases enrolled for the study (*N*—number of samples, %). For healthy controls in ovarian dataset, male samples were also used. As imPlatelet classifier is based on KEGG pathways, and not the entire transcriptome, observed TEP scores demonstrated that gender did not affect accuracy of classification in this particular case.

	NSCLC dataset		Sarcoma dataset			OC dataset		
	NSCLC	Healthy control	Sarcoma	Former sarcoma	Healthy control	OC	Benign control	Healthy control
	*N* = 401	*N* = 204	*N* = 62	*N* = 37	*N* = 75	*N* = 28	*N* = 30	*N* = 204
Median age (min‐max)	63 (27–88)	43 (18–86)	60 (27–78)	63 (31–83)	55 (21–76)	62.0 (36–83)	49.5 (24–81)	42.5 (18–86)
Females	164 (40.90%)	118 (58.13%)	28 (45.16%)	19 (51.35%)	75 (100%)	28 (100%)	30 (100%)	119 (58.33%)
Stage I	57 (14.21%)	NA	7 (11.29%)	NA	NA	4 (1429%)	NA	NA
Stage II		NA	1 (1.61%)	NA	NA	5 (17.86%)	NA	NA
Stage III		NA	9 (14.51%)	NA	NA	14 (50.00%)	NA	NA
Stage IV	343 (85.54%)	NA	44 (70.97%)	NA	NA	2 (7.14%)	NA	NA
Unknown	1 (0.25%)	NA	1 (1.61%)	NA	NA	3 (10.71%)	NA	NA

Blood collected in Gdańsk was processed within 48 h upon collection, strictly according to thromboSeq protocol [22]. Briefly, tubes were centrifuged to obtain platelet‐rich plasma (20 min, 120 g), pelleted (20 min 360 g), and carefully resuspended in RNAlater (Thermo Scientific, Waltham, MA, USA). After overnight incubation at 4 °C, platelets were stored at −80 °C. Blood collected in Gdańsk was shipped to Cancer Center Amsterdam, VU University Medical Center for further processing. RNA was extracted with mirVana miRNA isolation kit (Ambion, Thermo Scientific). For RNA quality verification, the RNA 6000 Pico Chip (Bioanalyzer 2100; Agilent, Waltham, MA, USA) was used. Subsequently, SMARTer Ultra Low RNA Kit for Illumina Sequencing v3 (Clontech, Mountain View, CA, USA) was used for cDNA amplification. Next, cDNA was subjected to nucleic acid shearing by sonication (Covaris Inc., Woburn, MA, USA) and then labeled with single index barcodes for Illumina sequencing using the TruSeq Nano DNA Sample Prep Kit (Illumina, San Diego, CA, USA). Labeled platelet cDNA library was measured with the DNA 7500 chip or DNA High Sensitivity chip (Agilent). High‐quality samples were pooled and submitted for 100 bp Single‐Read sequencing on the Illumina HiSeq 2500 and HiSeq 4000 platform.

### 2.2 Data preparation

Raw RNA‐seq data encoded in FASTQ‐files were subjected to a standardized RNA‐seq alignment pipeline, as described in thromboSeq protocol [22]. Briefly, reads were subjected to trimming of sequence adapters by trimmomatic (v. 0.22) https://www.ncbi.nlm.nih.gov/pmc/articles/PMC4103590/, mapped to the human reference genome (hg19) using star (v. 2.3.0), and summarized using htseq (v. 0.6.1), guided by the Ensembl gene annotation version 75. The expression data for each sample were normalized using DESeq2 package [26] with variance‐stabilizing transformation [27]. Gencode v19 GRCh37 annotation [28] was used for annotation. Importantly, samples with < 100 000 total reads were excluded from further analysis. The cutoff was determined arbitrarily as a compromise between the number of samples available and quality of samples. Ensemble IDs were converted into gene names using Gencode v19 GRCh37 annotation [28]. Only genes with gene status known were used. If two IDs were mapped to the same gene name, expression data for ID marked in Gencode Gene Transfer Format as level 1 were used. Transcripts that could not be mapped to a transcript with Gencode status ‘known’ were excluded. Filtered expression profiles were then used to build images, where each pixel corresponded to the expression level of a certain gene. As each sample consisted of 39 843 splice variants, we decided to arrange pixels. As our classifier was developed to recognize TEPs of cancer patients, we decided to especially focus on pathways which might be deregulated due to tumor development. We visited KEGG database [23] and selected signaling pathways corresponding to three aspects: cancer, metabolism, and signaling processes. Combining these three groups of pathways resulted in higher accuracy than using just pathways marked as directly related to cancer. R package gage was used to gather KEGG pathway data [29]. In each pathway, KEGG IDs which were not linked to the expression level of a particular gene were removed from the corresponding row.

### 2.3 Deep neural network

Deep neural network (DNN) model was built using keras r package [30] with TensorFlow 2.0 backend [31]. It consisted of 10 layers, including eight hidden layers: two 2‐dimensional convolutional layers, each with four filters and kernel size of 3 × 3, four densely connected layers with gradually reduced number of units, and two dropout layers. Binary cross‐entropy was used as a loss function. Gradient optimization was performed using adadelta algorithm [32]. Since the dataset was imbalanced, classes received weights proportionate to their frequencies. To further improve sensitivity, the weight of OC cases was increased by an additional scaling factor of 1.06 (determined experimentally). Training data were shuffled before each epoch using keras built‐in functionality. Hyperparameters of the model were selected experimentally.

### 2.4 Model validation

In OC, independent test set consisted of 40% of stratified random samples. Remaining 60% of the samples were used for stratified five‐fold cross‐validation, with four subgroups used for training of the neural network and one subgroup used for the built‐in keras validation. Class balance was preserved in each of the subsets, including split of controls into healthy donors and benign controls used in OC classification. In sarcoma and NSCLC datasets, samples followed the splits from original articles [24, 25], with minor differences caused by modified quality control process. Each model was then tested using independent test set. Assignment of samples to each data subset is presented in Table S1. Cross‐validated area under curve (AUC) was computed using cvAUC r package [33]. Cross‐validated receiver operating characteristic (ROC) curves were generated using ROCIT r package [34].

## 3 Results

### 3.1 Method overview

**Fig. 1:**
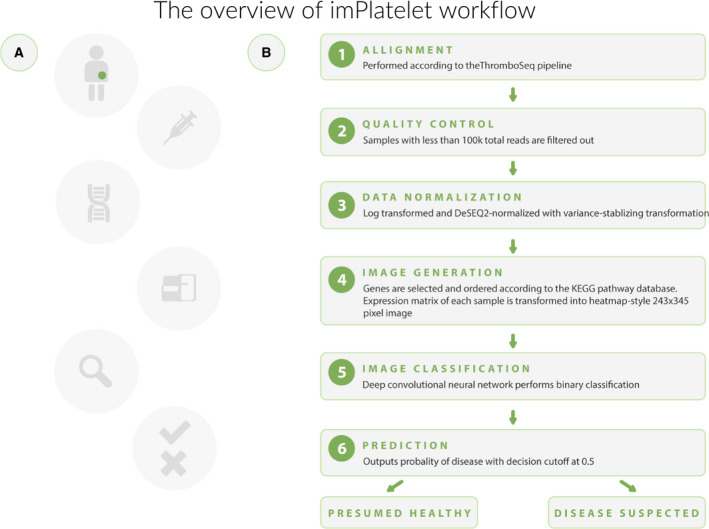
Overview of the imPlatelet workflow. (A) An overview of the developed approach. A blood sample is collected from a patient with a suspicion of cancer; isolated platelets are subjected to RNA extraction and sequencing; and read counts are normalized, followed by image generation that is recognized by the DNN. (B) An overview of DNN‐based method development, with a focus on sequencing data processing.

**Fig. 2:**
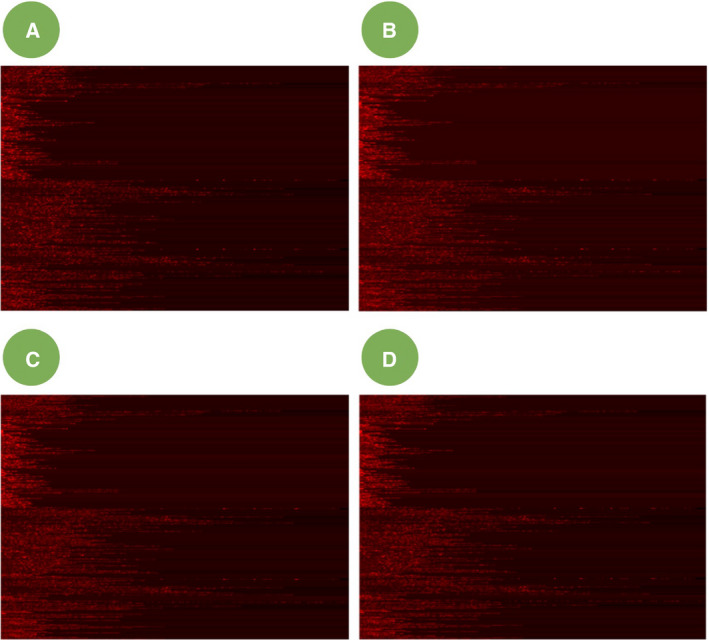
Platelet RNA counts are converted into images representing pathway expression profiles. Images of the following samples are shown: (A) TR1882 (healthy control), (B) TR2199 (NSCLC), (C) TR4550 (sarcoma), and (D) TR4379 (OC).

In this work, we present imPlatelet—a new classification method which accurately recognizes platelet transcriptome, utilizing deep learning. The method is a part of the liquid biopsy protocol, focused on the analysis of the sequencing data from platelet RNA (Fig. 1A and Movie S1) [22]. This methodology has been developed to further improve the classification accuracies over the currently employed PSO‐enhanced SVM algorithm. It is based on the hypothesis that due to external queues, platelet pre‐mRNA is subjected to splicing in a pathway‐specific manner. Our approach introduces two additional steps to the gold standard RNA data classification procedure, namely pathway panel construction and image‐based classification. After standard preprocessing (Fig. 1B, steps 1–3), numeric expression values from a sample are turned into an image—a pathway panel (Fig. 2). Rows of the image represent KEGG cancer‐related pathways, pixels represent particular genes in the pathways, while pixel intensity represents the expression of a specific gene. Importantly, one splice variant can occur in the image multiple times, if it is relevant in several pathways (i.e., splice variants encoding MAPK pathway elements or cyclins—crucial elements in cancer progression). Once the images are generated, the samples are subject to image‐based classification using DNN. The network is trained using a subset of samples and tested in an independent cohort. Compared with standard methods (e.g., SVM, random forest, and other previously published), it additionally accounts for gene co‐expression and gene‐to‐gene interactions in a group of preselected pathways.

### 3.2 Panel construction

RNA‐based diagnostics can benefit from gene panels, similarly to DNA‐focused studies. We present here a cancer panel based on KEGG Pathway database [23], applied to TEPs. The panel consists of 243 pathways extracted from KEGG database: 22 pathways directly implicated in cancer, 131 signaling‐related pathways, and 90 pathways involved in metabolism (Table S1). The pathways were ordered according to cancer, metabolic, and signaling pathways delineated in KEGG. Ordered and normalized spliced RNA transcripts of each sample were used as input to generate a color image, unique for each sample. Image created for cancer panel consists of 243 rows (pathways) and 345 columns (length of the longest pathway). The spectrum of pixel intensities varies from black (no spliced RNA read counts) to red (high spliced RNA read counts)—examples are presented in Fig. 2.

### 3.3 Classification using deep neural network

Panel approach transforms numeric RNA‐sequencing data into a set of figures. Each input sample is turned into a separate image. Therefore, widely used classification methods like SVM or random forests cannot be utilized for this type of input. To classify the generated image data, we employed a DNN, based on TensorFlow and Keras libraries, implemented in r [35].

**Fig. 3:**
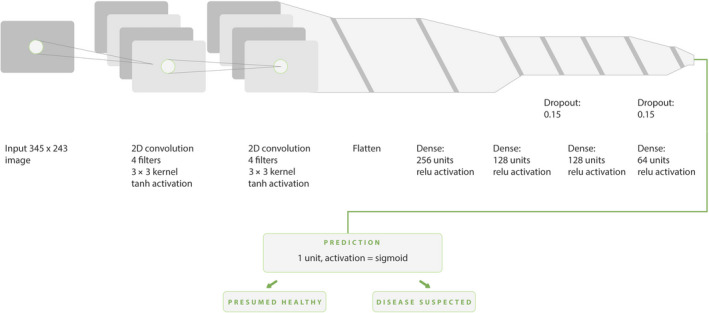
Structure of the DNN illustrated using NN‐SVG tool (http://alexlenail.me/NN‐SVG/ LeNet.html). The figure illustrates how the images are processed through convolution with hyperbolic tangent activation (tanh), hidden layers of rectified linear units (relu), and dropout thresholds. The classification process with sigmoid function delivers a binary output of the classification (presumed healthy or disease suspected).

In our approach, the network resembles visual cortex of the brain, characterized by a high level of connectivity. The DNN (Fig. 3) consists of convolution, flatten and hidden layers. First, application of the convolution layer transforms the images into a feature map, defined by a number of images, map width and height, and feature channels. After convolution, the stream of data is flattened to a one‐dimensional array of elements. Next, a hidden layer with rectified linear units is the highly connected part which is trained in a similar way to human memory, using train data. The dropout layers are additionally introduced in the hidden layers to avoid overfitting.

Once the network is trained using the input dataset, a test set of independent samples (not used in the training procedure) is classified. The final sample class decision is conducted using the output unit, activated by a sigmoid function. Because of the sigmoid function application, our method currently supports only two‐class decision making.

### 3.4 Performance of the method

To evaluate imPlatelet, we tested the method in three TEP datasets. Sample selection was preceded by quality control filtering. The first dataset consisted of 401 NSCLC and 204 healthy donor samples [24, 36], the second dataset consisted of 62 sarcoma samples, 37 former sarcoma samples, and 75 matched healthy donor samples [25], while the third, unpublished dataset included platelets collected from 28 OC patients and 30 benign control samples (cysts, Brenner tumors, cystadenofibromas which sometimes resemble OC, and other ailments such as mature teratomas, myomas of the uterus, adenomyoses or leiomyomas), five patients with borderline tumors and four nonstandard OC cases (relapses, OC after surgery but prior to chemotherapy administration). For the training purposes, only OC cases, benign controls, and healthy controls were used. Summary is presented as Tables S2 and S3. Raw files are deposited at Gene Expression Omnibus, under the accession number GSE158508 [37].

**Fig. 4:**
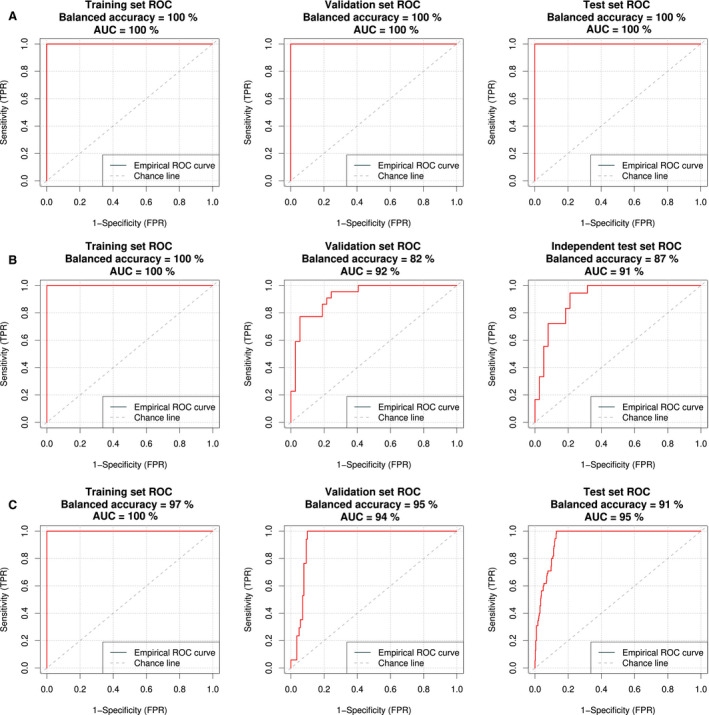
Cross‐validated ROC curves for the training set (left), validation set (middle), and the independent test set (right) in (A) NSCLC dataset (healthy donors *N* = 203, NSCLC cases *N* = 401); (B) sarcoma dataset (healthy donors and former sarcoma *N* = 112, sarcoma cases *N* = 62); and (C) OC dataset (healthy donors and benign control samples *N* = 233, OC cases *N* = 28).

The NSCLC dataset included mostly stage IV cases (343/401). As the advanced disease strongly affects the platelet expression profile, the performance of the classifier in this dataset was excellent. Specificity, sensitivity, and balanced accuracy were all equal to 100% (Fig. 4A, Table 1), as opposed to 99% (training set) and 98% (validation set) in the unmatched cohort reported by Best *et al*. [24]. Note, that in work of Best *et al*., validation set was called evaluation set, and independent test set was called validation set.

Sarcoma dataset was more challenging in classification than NCSLC dataset. First, the number of samples was lower and the dataset was imbalanced (62 cancer cases versus 112 healthy donor and former sarcoma cases collectively). Second, patients had less advanced disease than NSCLC cohort (stages ranging from I to IV, with 44 out of 62 classified as stage IV). Both of these factors made DNN training and testing more complicated (Fig. 4B). We focused on the balanced accuracy instead of standard AUC of classification, to account for sample size differences in the affected and healthy group. Our method reached 87% of balanced accuracy in an independent test set (Table 1) when compared with 84% balanced accuracy calculated for the independent test set results presented by Heinhuis *et al*. [25]. We also observed an improved sensitivity for samples classified with imPlatelet: 94%, compared to 88% reported for the independent test set in thromboSeq method [25].

**Fig. 5:**
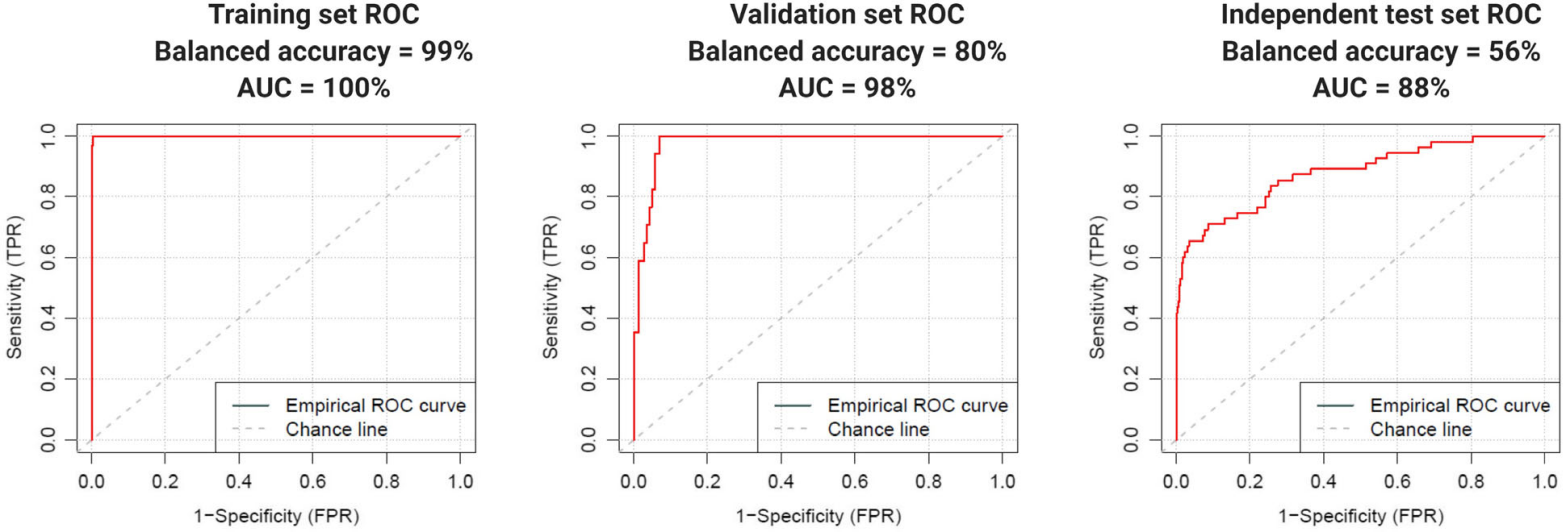
Cross‐validated ROC curves for the training set (left), validation set (middle), and the independent test set (right) in OC dataset, calculated with thromboSeq.

Finally, imPlatelet method was tested in our own, previously unpublished OC dataset (Fig. 4C). In an independent test set, imPlatelet reached 91% balanced accuracy, with sensitivity and specificity equal to 95% and 88%, respectively (Table 2). Despite the small sample size, generated ROC curves would show high performance with AUC equal to 0.95 in the independent dataset (Fig. 4C), showing small standard deviation and narrow 95% CI range (Table 1). To further verify the performance of the developed method, we recreated the ROC curves, using thromboSeq protocol (Fig. 5). This time the performance would reach 56% balanced accuracy, compared to 91% reached by imPlatelet. Confusion matrices for the training, validation, and the independent test sets are presented as Table S4).

**Table 2:** Performance of imPlatelet classification in the used datasets (NSCLC, sarcoma, and OC). Sensitivity and balanced accuracy in an independent test set are in bold. Some values of 1 are in fact round‐offs of 0.999 values.

	NSCLC and controls (healthy donors) *N* = 605			Sarcoma and controls (healthy donors and former sarcoma) *N* = 174			OC and controls (healthy donors and benign controls) *N* = 262		
	Mean	SD	95% CI	Mean	SD	95% CI	Mean	SD	95% CI
Training set									
Sensitivity	100%	NA	NA	100%	NA	NA	100%	0	100–100%
Specificity	100%	NA	NA	100%	NA	NA	94%	2	92–96%
Balanced accuracy	100%	NA	NA	100%	NA	NA	97%	1	96–98%
Precision	100%	0	NA	100%	NA		66%	5	60–73%
Recall	100%	0	NA	100%	NA		100%	0	100–100%
Specificity at 100%	100%	NA	NA	100%	NA	NA	100%	0	100–100%
AUC	100%	0	100–100%	100%	0	100–100%	100%	0	100–100%
Validation set									
Sensitivity	100%	NA	NA	86%	NA	NA	100%	0	84–100%
Specificity	100%	NA	NA	78%	NA	NA	90%	2	88–92%
Balanced accuracy	100%	NA	NA	82%	NA	NA	95%	1	94–96%
Precision	100%	NA	NA	70%	NA	NA	55%	5	49–61%
Recall	100%	NA	NA	86%	NA	NA	100%	0	100–100%
Specificity at 100%	100%	NA	NA	59%	NA	NA	87%	0.02	89–94%
AUC	100%	0	100–100%	92%	3	86–99%	94%	0.02	90–98%
Independent test set									
Sensitivity	100%	NA	NA	94%	NA	NA	95%	8	84–100%
Specificity	100%	NA	NA	79%	NA	NA	88%	1	87–89%
Balanced accuracy	100%	NA	NA	87%	NA	NA	91%	4	87–96%
Precision	100%	0	100–100%	68%	NA	NA	48%	2	45–50%
Recall	100%	0		94%	NA	NA	95%	8	84–100%
Specificity at 100%	100%	NA	100–100%	68%	NA	NA	87.5%	0	87–88%
AUC	100%	0	100–100%	91%	4	84–98%	95%	1	93–97%

**Fig. 6:**
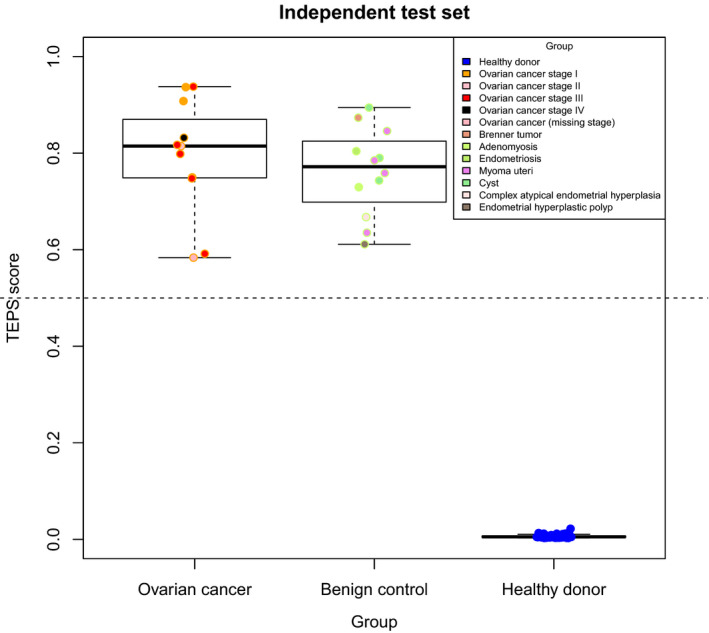
Distribution of platelets (TEP score) for the independent set, calculated with imPlatelet. Three discriminative cohorts were included: patients with OC, gynecological patients with conditions other than OC (benign controls), and healthy donors. Samples are colored by their cancer stage (OC) or their final diagnosis (benign controls). Scores are average values after a sample five‐fold cross‐validation run. Independent set consisted of 11 OC patients, 12 benign gynecological patients, and 81 healthy controls. Boxes contain samples with TEP scores between Q1 and Q3 of respective group, and the thick lines represent medians of each set. Whiskers mark minimum and maximum scores in each set.

**Fig. 7:**
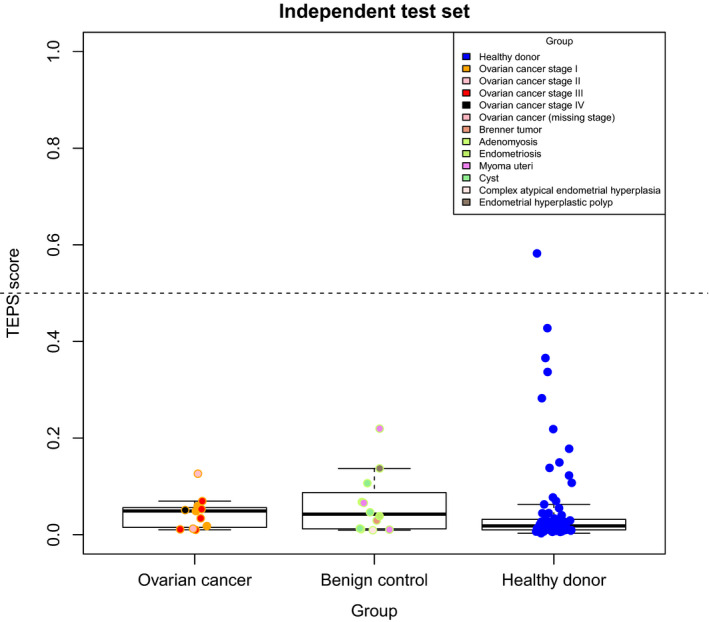
Distribution of platelets (TEP score) for the independent set, calculated with thromboSeq. Three discriminative cohorts were included: patients with OC, gynecological patients with conditions other than OC (benign controls), and healthy donors. Samples are colored by their cancer stage (OC) or their final diagnosis (benign controls). Scores are average values after a sample five‐fold cross‐validation run. Independent set consisted of 11 OC patients, 12 benign gynecological patients, and 81 healthy controls. Boxes contain samples with TEP scores between Q1 and Q3 of respective group, and the thick lines represent medians of each set. Whiskers mark minimum and maximum scores in each set.

Next, in order to provide better insight in the classification dynamics of patients with OC, benign controls and healthy donors, we generated the distribution of the TEP scores in samples belonging to the independent set, classified with imPlatelet and thromboSeq, respectively (Figs 6 and 7). TEP scores are classifier outputs which are nonbinary. TEP score equal to 0 indicates a healthy donor, whereas TEP score close to 1 indicates a cancer case. The value is an indirect measure of probability of a sample being a cancer sample. While healthy donors would always classify correctly, there was an overlap between OC samples and benign controls, the latter showing elevated an elevated CA‐125 level in roughly 40% of cases.

## 4 Discussion

Our imPlatelet classifier reached higher balanced accuracy and sensitivity in all three tested independent datasets: lung cancer, sarcoma, and OC. So far, no data have been published on TEPs in OC. Liquid biopsy alternatives, namely CTCs and ctDNA, proved limited utility in the early diagnosis of the disease. Detection of CTCs in OC is difficult even in the advanced stages of the disease [38], and the analysis of ctDNA has limited application in cancer detection. Studies on ctDNA in OC demonstrated relatively high specificity (> 88%), with variable sensitivity (27–100%) [38]. Importantly, tests such as CancerSEEK or TEC‐Seq would reach ~ 97% sensitivity and > 99% specificity, but these studies relied on healthy individuals [39, 40], contrary to benign controls included in our study. Furthermore, the median sensitivity of CancerSEEK would drop to 73% for stage II cancer [39].

According to our knowledge, this is the first study implementing an image‐based approach to classify biological samples. While works of Matsubara *et al*., Aliper *et al*., and Antonio *et al*. rely on deep learning, the authors focus on data other than RNA sequencing and the architecture of their network differs considerably [41, 42, 43]. In the current study, we show high performance of imPlatelet method applied to cancer sample classification. Despite the early stage, many OC samples would still classify correctly in most of cross‐validations. High accuracy, despite the low number of cases, is partially owed to biological knowledge implemented into the algorithm. Integrating insight of KEGG Pathway Encyclopedia adds an additional layer of input data, arranging all the analyzed splice variants according to their biological context, thus providing the level of details unattainable before. Eventually, not only the splice variants alone, but also their mutual codependence is taken into account.

Important features of imPlatelet classifier are the calculation of balanced accuracy and execution time. Some methods tend to classify all samples as cases or controls in an unbalanced dataset, leading to performance estimation bias. Balanced accuracy is therefore suitable also for datasets with a small number of cases. In terms of execution time, the training process in the DNN‐based approach takes minutes, up to hours, as opposed to thromboSeq which requires days, up to weeks. five‐fold cross‐validation of the model with OC data takes approximately 6 min, compared to over 3 days using thromboSeq. Once the model is trained, the testing procedure in imPlatelet requires less than one second per sample, using a desktop computer (CPU: Intel i‐7 9700k, RAM: 64 GB, GPU: GeForce GTX 1060 6GB). Consequently, for diagnostic use, imPlatelet could be executed on a laptop with an attached tensor processing unit accelerator.

Several limitations of the study need to be acknowledged. First, we had a limited number of OC cases when compared with nonmalignant and healthy controls. The recently published studies suggest that even 70–80 cases are a reasonable number [25, 44], but ideally we would prefer to work on hundreds of samples. These are very likely to be available in the near future. Another limitation was control samples belonging to volunteers of different age and sex. However, platelet transcriptome remains relatively consistent in healthy controls and all healthy samples would classify correctly, irrespectively of these two variables. As much as from human perspective, it might be difficult to discriminate between the case and the control samples; contrary to human perception, DNN identifies patterns and relations in panel images, resulting in high classification accuracy.

Summing up, our developed algorithm shows superior performance, detecting cancer cases even in the early I and II stage OC. It shows remarkable potential in healthy individuals' indication, paving the way to correct classification in other diseases. We believe that similar approach could be applied to sequencing data in tissues. For example, analysis of splice variants in tumor tissue has been considered promising in cancer immunotherapy [45]. In the field of liquid biopsy, imPlatelet could hopefully be adapted to studying cells other than single tumor cells, as the disease also affects other cells' expression profiles, for example, NK or T lymphocytes [46]. For the future projects, our DNN‐based classification could benefit from including additional panels, such as KEGG signaling pathways associated with the immune system or drug development. Another option could be the introduction of integrative omics analysis, with a panel including both splice variant and protein expression data, as reported by Mantini *et al*. [47]. Furthermore, even though imPlatelet yields typically binary output, the method could be adapted to multiclass classification.

## 5 Conclusions

To our knowledge, this is the first report that uses a DNN to analyze RNA‐sequencing data. We believe we have created a classifier which significantly outperforms other available tools used in cancer diagnostics: GRAIL [48], CancerSEEK [39], and, previously published by our collaborators, thromboSeq [22]. The developed algorithm shows superior performance, detecting cancer cases even in the early OC, partially owing its accuracy to another layer of information on the biological significance of RNA reads—the KEGG. The classifier's remarkable potential to indicate healthy individuals paves the way to the correct classification of other diseases.

## Code availability

Code available at https://github.com/KrzysztofPastuszak/Implatelet

## Author contributions

KP conceptualized the study, performed methodology, involved in software programming, validated the study, performed formal analysis, involved in investigation, provided resources, curated the data, wrote, reviewed, and edited the draft, and visualized the data. AS conceptualized the study, performed methodology, involved in investigation, provided resources, curated the data, wrote, reviewed, and edited the draft, visualized the data, supervised the study, involved in project administration, and acquired the funding. MGB conceptualized the study, involved in investigation, provided resources, curated the data, and wrote, reviewed, and edited the draft. SGJGIV curated the data and wrote and reviewed the draft. SŁS conceptualized the study, involved in investigation, provided resources, curated the data, and wrote, reviewed, and edited the draft. AŁ provided resources, curated the data, and wrote and reviewed the draft. RR conceptualized the study, performed methodology, involved in investigation, and wrote, reviewed, and edited the draft. AJŻ conceptualized the study, provided resources, wrote, reviewed, and edited the draft, and acquired the funding. JJ conceptualized the study, involved in investigation, provided resources, wrote, reviewed, and edited the draft, supervised the study, and acquired the funding. TW conceptualized the study, involved in investigation, provided resources, curated the data, wrote, reviewed, and edited the draft, and acquired the funding. TS conceptualized the study, involved in investigation, wrote and reviewed the draft, and supervised the study.

## Conflict of interest

TW and MGB are inventors on relevant patents. TW is shareholder of GRAIL Inc. KP, AS, TS, AJZ, and JJ have filed a patent application regarding the classifier.

### Peer Review

The peer review history for this article is available at https://publons.com/publon/10.1002/1878‐0261.13014

## Supporting information

**Table S1**. The table summarizes the panel of 243 pathways extracted from KEGG database: 22 pathways directly implicated in cancer, 131 signaling‐related pathways and 90 pathways involved in metabolism.
**Table S2**. The table cases used for training, validation and independent test set. For sarcoma dataset, controls include healthy donors and former sarcoma samples. For OC dataset, controls include benign controls and healthy donor samples.
**Table S3**. The table specifies which cases were used for which experiment. OC used 5‐fold cross‐validation with independent test set. Both sarcoma and NSCLC experiments were conducted using sample splits as close as possible to those used in the original articles. Several samples from the NSCLC and sarcoma datasets were not included as they did not meet the QC requirements.
**Table S4**. Performance of thromboSeq on the same dataset that was used for OC classification by imPlatelet.

**Movie S1**. Manuscript overview. The link to the promo video can be found here: https://youtu.be/FrPFehYQHLU.

## Data Availability

Raw files are deposited at Gene Expression Omnibus, under the accession number GSE158508.
