# Supplementary material for: imPlatelet classifier: image‐converted RNA biomarker profiles enable blood‐based cancer diagnostics
Source: Mol Oncol. 2021 Jun 20;15(10):2688–701. doi: 10.1002/1878-0261.13014 (PMC8486571; doi:10.1002/1878-0261.13014)
Supplement: Supplementary file 2 — Movie S1. Manuscript overview. The link to the promo video can be found here: https://youtu.be/FrPFehYQHLU. [file MOL2-15-2688-s002.docx]

**Supplementary Material**, **Movie S1** Manuscript overview. The link to the promo video can be found here: https://youtu.be/FrPFehYQHLU.

Title:

imPlatelet classifier: Image-converted RNA biomarker profiles enable blood-based cancer diagnostics

Authors:

Krzysztof Pastuszak^1,2^‡, Anna Supernat^1^*‡, Myron G Best^3,4,5^, Sjors G J G In ‘t Veld^3,4^, Sylwia Łapińska-Szumczyk^6^, Anna Łojkowska^6^, Robert Różański, Anna J Żaczek^1^, Jacek Jassem^7^, Tom Würdinger^3,4^, Tomasz Stokowy^8^
